# Sharpening emitter localization in front of a tuned mirror

**DOI:** 10.1038/s41377-018-0104-z

**Published:** 2018-12-05

**Authors:** Hannah S. Heil, Benjamin Schreiber, Ralph Götz, Monika Emmerling, Marie-Christine Dabauvalle, Georg Krohne, Sven Höfling, Martin Kamp, Markus Sauer, Katrin G. Heinze

**Affiliations:** 1 Rudolf Virchow Center, Research Center for Experimental Biomedicine, University of Würzburg, Josef-Schneider-Str.2, 97080 Würzburg, Germany; 2 Department of Biotechnology and Biophysics, Biozentrum, University of Würzburg, Am Hubland, 97074 Würzburg, Germany; 3 Technische Physik, Physikalisches Institut and Wilhelm Conrad Röntgen-Center for Complex Material Systems, University of Würzburg, Am Hubland, 97074 Würzburg, Germany; 4 Division of Electron Microscopy, Biozentrum, University of Würzburg, Am Hubland, 97074 Würzburg, Germany; 5 SUPA, School of Physics and Astronomy, University of St Andrews, St Andrews, KY16 9SS UK

## Abstract

Single-molecule localization microscopy (SMLM) aims for maximized precision and a high signal-to-noise ratio^1^. Both features can be provided by placing the emitter in front of a metal-dielectric nanocoating that acts as a tuned mirror^2–4.^ Here, we demonstrate that a higher photon yield at a lower background on biocompatible metal-dielectric nanocoatings substantially improves SMLM performance and increases the localization precision by up to a factor of two. The resolution improvement relies solely on easy-to-fabricate nanocoatings on standard glass coverslips and is spectrally and spatially tunable by the layer design and wavelength, as experimentally demonstrated for dual-color SMLM in cells.

Concepts for mirror-enhanced fluorescence have been around for centuries. In the 1970s, fluorophore-metal interactions were studied in depth^5^, followed by the development of a quantitative theory based on semiclassical quantum mechanics^6^. For an emitter located in the vicinity of a metal-dielectric substrate, the metal surface acts as a mirror, which leads to an enhancement and modulation of the excitation field^2^, the fluorescence decay rates and the quantum yield^3,6^ that arise from interference effects, and an enhanced detectability due to virtual 4Pi fluorescence detection^4^. Mirror-enhanced concepts have been shown to be compatible with superresolution modalities^7;^ however, their combined strengths have not yet been employed to their full potential.

SMLM methods excel in visualization of the cellular architecture at a molecular level^1^. The common concept of all SMLM methods is the separation of the fluorescence emission of individual fluorophores in time by photoactivation and photoconversion^8^, photoswitching^9,10^, or transient binding^11^, with subsequent determination of the single fluorophores’ position and image reconstruction. Thus, SMLM is able to push the resolution to ~20 nm in the lateral direction without further tweaks and tricks. The crucial parameter that determines the final resolution is the localization precision, which mainly depends on the number of fluorescence photons detected per localization event^12^. Several attempts to improve the localization precision have been reported, including optimized fluorescent dyes^13^, additives^14^, cryo-methods^15^, and 4Pi-microscopy^16^. Unfortunately, most approaches lack remarkable improvements or result in further limitations concerning complexity or compatibility with live cells.

As we show here, quenching and enhancement effects in the vicinity of metal-dielectric nanocoatings can be used to enhance contrast by suppressing background noise and improving the photon yield of the fluorophores. Easy-to-fabricate biocompatible metal-dielectric nanocoatings on glass coverslips can substantially improve the localization precision of *direct* stochastic optical reconstruction microscopy (*d*STORM) by a factor of two using a standard epifluorescence setup, which still exceeds the performance of *d*STORM using total internal reflection microscopy (TIRFM).

First, mirror-enhanced *d*STORM is demonstrated for the nuclear pore complex (NPC), which plays a key role in the regulation of molecular traffic between the cytoplasm and the nucleus^17^. Various superresolution microscopy studies have demonstrated their capability to resolve the eightfold symmetry of the NPC^18,19^. To identify the ideal layer design for mirror-enhanced *d*STORM of NPCs and to match the enhancement range to the fluorophore’s height range above the coverslip, we performed finite element method simulations of the distance-dependent excitation and emission enhancement for the fluorophore of choice (Fig. 1a), Alexa Fluor 647 (A647). Labeling the pore anchoring protein gp210 by classical immunolabeling, the fluorophores are expected at a distance of ~50 nm above the coverslip. To selectively enhance the emission in this height region, the optimal coating design features a 2 nm germanium (Ge) layer, followed by a 50 nm silver (Ag) layer covered by 10 nm of silicon nitride (Si_3_N_4_) (Fig. 1a). The maximum axial extension of the enhancement window (~120 nm) is wavelengths-dependent^20^, comparable to those reached by other evanescent techniques such as TIRFM, and thus powerful for selective imaging of membrane proteins in adherent cells.

**Fig. 1:**
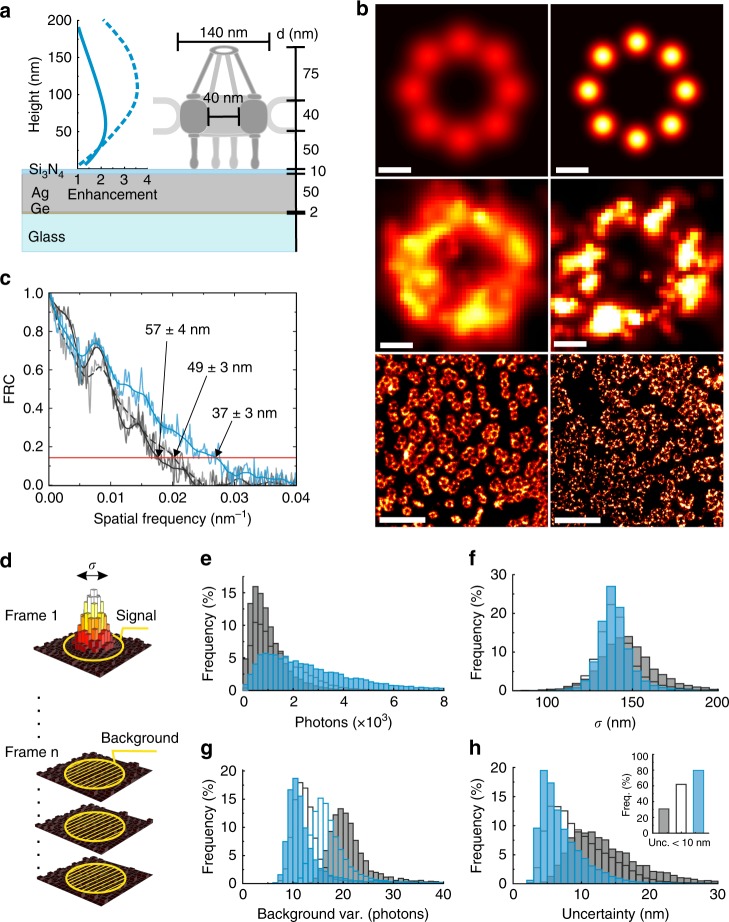
Resolving the NPC. **a** Optimized metal-dielectric substrate designed to exhibit the strongest enhancement field for emission (solid line) and excitation (dashed line) of the red emitting dye A647 at the axial position of the NPC ring structure labeling the pore anchoring protein gp210. **b** Simulated conventional (localization precision: 20 nm, left) and mirror-enhanced *d*STORM (12 nm, right) images; respective experimental image of a single NPC ring and overview images. **c** Fourier ring correlation (FRC) resolution estimation of sunny-side-down (gray solid line, see Supplementary Fig. S1), TIRF- (gray dashed line) and mirror-enhanced *d*STORM (blue line) images of **b**. **d–h** Statistical analysis. **d** Principle of TRABI^22^. Histograms of **e** intensity distribution, **f** standard deviation, **g** background variance, and **h** resulting localization uncertainty of the localization events for coated coverslips (blue) with half (filled bars) and the same (open bars) excitation power as in the experiments on uncoated coverslips (gray) in the sunny-side-down (filled bars) and TIRF configurations. The inset in **h** highlights the increased number of events with a localization uncertainty below 10 nm for coated (blue) versus uncoated coverslips in the sunny-side-down (gray filled bars) and TIRF (gray open bars) configurations. Scale bars: 50 nm (single rings) and 1 µm (overview)

Assuming that the eight gp210 proteins per NPC would be labeled with a single A647 and a localization precision of *σ* ≈ 20 nm, the eight elements of the NPC ring would appear as spatially overlapping signals in a simulated *d*STORM image (Fig. 1b). With a predicted two-fold fluorescence enhancement by metal-dielectric coatings (Fig. 1a), the resolution could be substantially improved (Fig. 1b). To test this enhancement experimentally, we performed mirror-enhanced *d*STORM experiments with nuclear envelopes spread on the metal-dielectric substrate and on a bare glass coverslip as a control sample. For the mirror-enhanced *d*STORM experiments, the nanocoating with the specimen faces the front lens of the water objective (NA 1.15) in a “sunny-side-down” (SSD) configuration. Control experiments on uncoated glass were performed in both the SSD (NA 1.15) and TIRF (oil objective, NA 1.46, see Supplementary Fig. S1) configurations. The acquisition conditions were the same for all experiments mentioned above except for the applied laser intensity. The excitation enhancement in mirror-enhanced *d*STORM by the mirror effect of the metal coating and the increased excitation intensity of the evanescent field in TIRF illumination allowed a 50% reduction of the laser intensity for both configurations, which still matches the photoswitching conditions of A647. The SSD *d*STORM image appears blurrier than the corresponding mirror-enhanced *d*STORM image, where the eight gp210 elements can be distinguished (Fig. 1b). An overall resolution enhancement of 150% was derived from Fourier ring correlation (FRC) analysis^21^ (Fig. 1c) based on the overview images (Fig. 1b). Importantly, the resolution of the mirror-enhanced *d*STORM image also exceeds that which can be achieved with TIRF *d*STORM by 25%.

To understand in more detail why mirror-enhanced *d*STORM provides sharper images, we analyzed the localization data by temporal, radial-aperture-based intensity estimation (TRABI)^22^. This photometric method determines the signal and noise levels independently of the data fitting model (Fig. 1d). Here, TRABI reveals that the intensity of a single localization event is increased two- to three-fold compared to that in the TIRF and SSD configurations (Fig. 1e). For all three configurations, the signal width is comparable (Fig. 1f), and the noise represented by the background variance is significantly reduced in the case of mirror-enhanced *d*STORM (Fig. 1g). Note that, in a typical *d*STORM experiment, the fluorophore is already excited at or close to the saturation level to ensure maximal photon emission during each on-event (Supplementary Fig. S2). Thus, a further increase in excitation intensity cannot result in brighter emission, but it can result in optical sectioning due to the height-dependent modulation of the enhancement field. Importantly, this optical sectioning excludes the first nanometers adjacent to the surface coating (Fig. 1a, peak at 60 nm) so that background noise is substantially reduced. This effective background suppression is induced by both the sectioning itself and the lower laser intensity, while the latter is fully sufficient to reach the optimal excitation rate.

Detailed analysis of each localization event revealed nearly identical reoccurrence numbers for *d*STORM versus mirror-enhanced *d*STORM, while the on-time duration and photon counts were increased for the latter (Supplementary Fig. S3). Consequently, the resolution benefit of mirror-enhanced *d*STORM originates from both the increased signal of each localization event and the increased on-time of the fluorophore in the on-state. Taken together, “more photons” and “less noise” eventually improve the localization uncertainty^12^ and thus the localization precision to <10 nm (Fig. 1h, inset graph). This effect was reproduced in independent experiments (Supplementary Fig. S1b–d). As the sample fabrication is very controllable and reproducible, the variation has to be attributed to the variation in the preparation of the nuclear membrane. Note that the enhancement can also be achieved for structures closer to the surface when the metal-dielectric layer thicknesses are adjusted accordingly. Imaging isolated microtubules represents a typical example that requires such low-distance surface imaging (Supplementary Fig. S4).

The option to selectively boost fluorescence at different heights makes mirror-enhanced *d*STORM highly suitable for tailored and improved investigations of membrane receptors or other cell membrane components. To experimentally show cell compatibility as well as spectral tunability of mirror-enhanced *d*STORM, we performed dual-color experiments on Jurkat T-cells to visualize the distribution of CD45 receptors. Cells were labeled with a 50:50 mixture of Alexa Fluor 532 (A532) and A647 anti-CD45 antibodies and imaged on metal-dielectric coated and non/coated glass coverslips (Fig. 2). Note that each CD45 (monomeric) receptor-linked protein tyrosine phosphatase molecule^23^ is labeled by only a single primary antibody carrying either A647 or A532 so that they cannot colocalize (Supplementary Fig. S5).

**Fig. 2:**
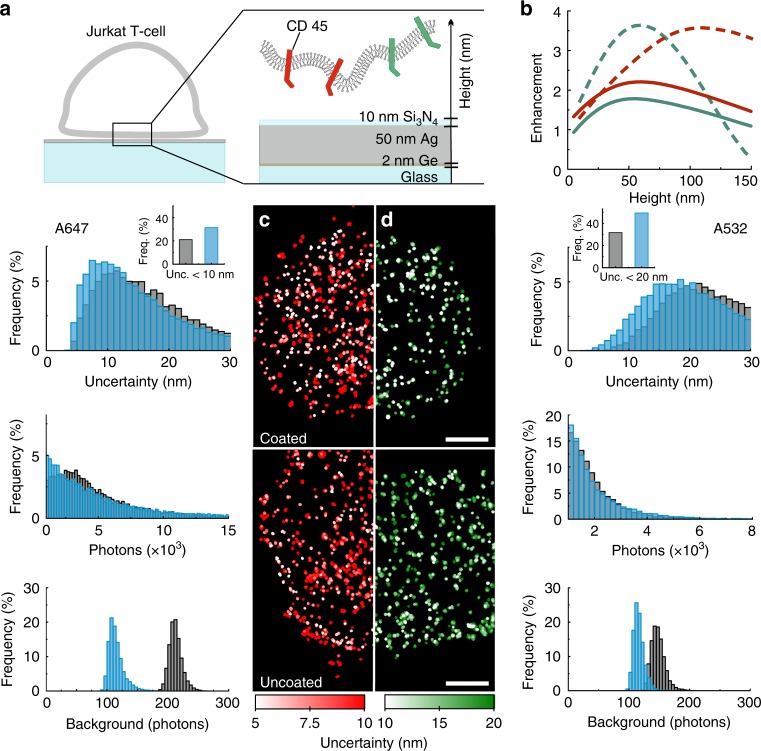
Dual-color mirror-enhanced *d*STORM of the CD45 receptor in immobilized Jurkat T-cells. **a** Experimental setup. **b** Simulation of excitation (dashed line) and emission (solid line) enhancement for A532 (green) and A647 (red). **c**, **d** Superresolution images and analysis: each CD45 (monomeric) receptor carries one color, either **c** A647 or **d** A532 and thus does not colocalize with its complementary stained counterpart. False-color images in red (**c**) and green (**d**) indicate the localization uncertainty per event for coated and uncoated coverslips; white spots indicate the highest precision. Histograms show the distributions of respective localization uncertainty, signal intensity, and background on uncoated (gray) and coated coverslips (blue). The inset highlights the increased number of events with a localization uncertainty **c** below 10 nm and **d** below 20 nm. Scale bars: 2 µm

Simulations on fluorescence enhancement for the two fluorophores of choice suggest that, while the excitation enhancement field is slightly shifted in height for the two fluorophores (Fig. 3a, b), the increase in detectability is comparable for both with a dominant contribution from the parallel dipole contributions (Fig. 3c, d). The resulting emission enhancement profile shows a slight shift in amplitude along the height axis as the quantum yield enhancement differs greatly due to the difference in intrinsic quantum yield of A532 (*η*_0_ = 0.61) and A647 (*η*_0_ = 0.33) (Fig. 3e, f).

**Fig. 3:**
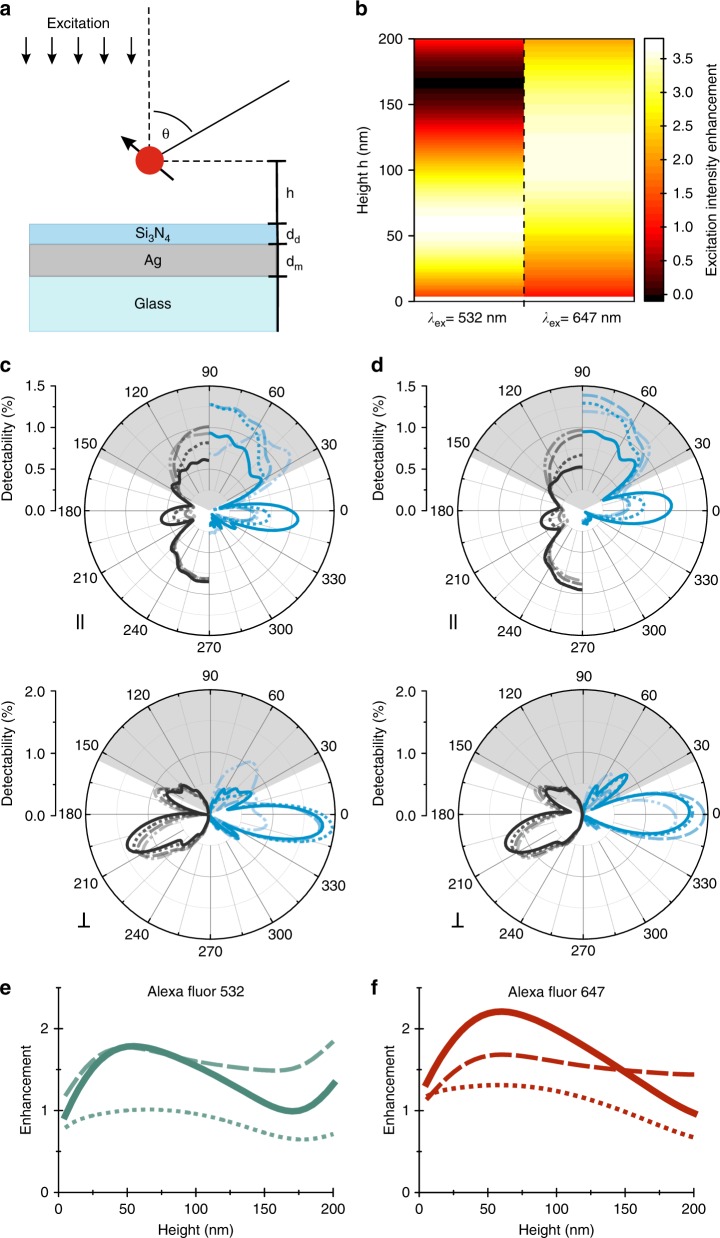
Simulation of the excitation and emission enhancement based on finite element method calculations. **a** Scheme of the sample geometry. **b** Excitation intensity enhancement in the vicinity of a silver nanocoating (*d*_m_= 50 nm, *d*_d_ = 10 nm) for two different excitation wavelengths (*λ*_ex_). **c**, **d** Far-field radiation patterns for parallel (II) and perpendicular (⊥.) dipole orientations in the vicinity of a glass coverslip (gray) and the silver nanocoating (blue) at a height of 10 nm (solid), 50 nm (doted), 100 nm (dashed), and 150 nm (dash-doted) for A532 (**c**) and A647 (**d**). **e**, **f** The combination of quantum yield enhancement (dotted) and detectability enhancement (dashed) leads to a tailored height-dependent emission enhancement profile (solid) for A532 (**e**) and A647 (**f**)

Nevertheless, both fluorophores can share an operating window. Thus, the simultaneous enhancement of spectrally distinct fluorophores with the same metal-dielectric coating design is feasible (Fig. 2b). Dual-color imaging confirms the spectral tunability and spatial selectivity of mirror-enhanced *d*STORM: false-color images indicate the localization uncertainty of events detected for each color on coated and uncoated glass coverslips for comparison with white spots, indicating higher localization precision (Fig. 2c, d). On coated coverslips, high-precision events are increased for both colors, resulting in improved image resolution. The histograms show the corresponding distributions of localization uncertainty, intensity and background variance for A647 and A532 (Fig. 2, left and right) on coated and uncoated coverslips. The graph insets (Fig. 2) depict events with localization uncertainties below 10 and 20 nm. For both colors, the occurrence of these localizations is increased by a factor of 1.5 due to higher brightness and background suppression.

Up to this point, all sample structures exhibited a planar architecture placing the features of interest directly in the enhancement region. In contrast, a three-dimensional (3D) sample will partly exceed the enhancement region so that features located in and outside the enhancement maximum can be distinguished within a very sharp height region. To deduce absolute height information based on the mirror-enhancement effect, we used 15 µm microspheres labeled with A647 as described by Cabriel et al.^24^. Based on the bead radius and center position, the axial position of each localization can be calculated (Fig. 4a). On the nanocoating (2 nm Ge, 50 nm Ag, and 10 nm Si_3_N_4_), the intensity and localization uncertainty show a clear height dependence with a broad maximum of the intensity at a height of 100 nm that translates to a minimum of localization uncertainty (Fig. 4b). The experimental axial intensity profile agrees well with the expected excitation and emission enhancement based on simulations (Fig. 4c). Notably, the simulated emission enhancement for higher distances of ~350 nm is fully compensated by a minimum of the excitation profile preventing an effective enhancement in this height region.

**Fig. 4:**
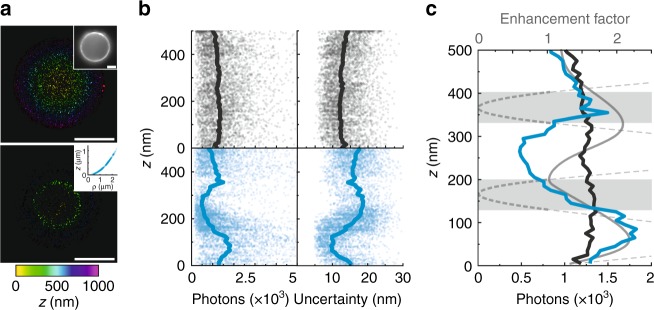
Axial calibration of mirror-enhanced *d*STORM. **a** Color-coded map of the z-position of A647 fluorophores labeling a 15 µm microsphere imaged in the SSD configuration (upper panel) and the mirror-enhanced dSTORM configuration (lower panel). The z-position was calculated based on the microsphere radius (diffraction-limited image of the equatorial plane, inset upper panel) and the radial position *ρ* of the single localization events respective to the bead center (inset lower panel). **b** Axial dependence of the intensity (left) and the localization uncertainty (right) of single events localized in the experiment in the SSD (upper graphs, gray dots) and the mirror-enhanced *d*STORM (lower graphs, blue dots) configurations. The solid lines mark the height-dependent average intensity and average localization uncertainty. **c** Comparison of the height-dependent intensity profile of the SSD (gray) and mirror-enhanced (blue) configurations with the simulated emission enhancement (solid light gray line) and excitation enhancement (dashed light gray line). The height regions in which the excitation enhancement drops below 1 are highlighted in light gray. Scale bars: 5 µm

This height-dependent profile can be translated into axial distances. Here, we demonstrate this for a 3D microtubule network of Cos7 cells where the average localization uncertainty serves as the axial ruler to pinpoint the height of single filaments (Fig. 5). For a conventional *d*STORM experiment, the localization uncertainty is consistent within a wide axial range (Fig. 5a), while there is a strong height dependence for mirror-enhanced *d*STORM. Filaments close to the surface, below ~130 nm, display the lowest localization uncertainty (Fig. 5b, green filaments) with a gradual increase in the localization uncertainty for filaments further above. This provides uncertainty-based image contrast that allows clear height distinction of crossing microtubules (Fig. 5b, c, crossing points marked by white arrows).

**Fig. 5:**
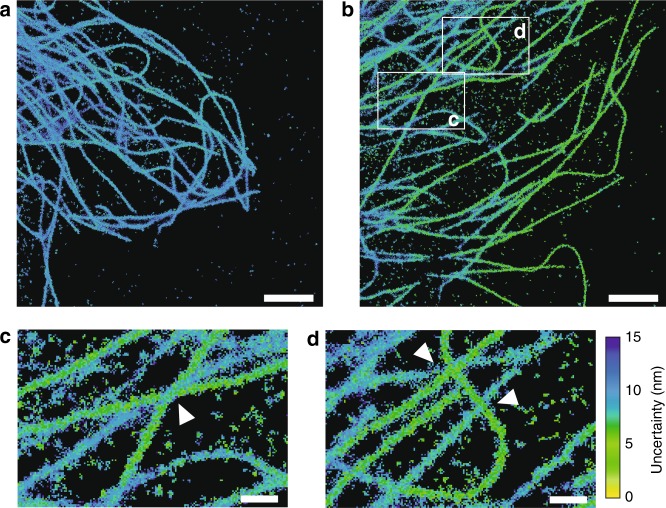
Resolving the 3D architecture of the cellular microtubule network with mirror-enhanced *d*STORM. **a**, **b** Scatter plot reconstructions of Cos7 microtubule networks immunolabeled with A647 and imaged with *d*STORM in **a** the SSD configuration and **b** the mirror-enhanced configuration. The color code illustrates the median of the localization uncertainty of all events localized in the respective pixel area. **c**, **d** Magnified image sections from **b**. The white arrows indicate microtubule crossings where different axial positions of the filaments are distinguishable based on the localization uncertainty. Scale bars: 2 µm (**a**, **b**) and 500 nm (**c**, **d**)

To summarize, metal-dielectric coatings are a versatile biophotonics tool that enable straightforward control of the axial fluorescence enhancement distribution by adjusting the distance of the fluorescent sample to the nanocoating or vice versa. The simple three-ply design of our coatings grants a straightforward one-step fabrication and allows tailoring the shape of the resulting enhancement field to the sample geometry and fluorescent label at hand. Coated coverslips can, in principle, be fabricated in tabletop thin-film deposition systems and be used in any SMLM setup without further training or caution and without the requirement of implementing TIRF illumination and a high NA objective. Furthermore, in contrast to TIRF approaches, mirror-enhanced SMLM allows highly controlled sectioning. For TIRF-based illumination, the penetration depth dramatically depends on the illumination angle, which is difficult to control in most common setups.

As experimentally demonstrated, the scope of applications for mirror-enhanced SMLM is comparable to traditional SMLM techniques enabling dual-color imaging of cells. Of course, the enhancement field of mirror-enhanced SMLM is bound to the surface and currently extends to 160 nm above the substrate interface. Moreover, single-molecule localization is still feasible without the boosting effect, as demonstrated here by interrogating the 3D microtubule architecture, and thus promotes mirror-enhanced SMLM as a 3D imaging tool. This is important as axial resolution is a bottleneck in SMLM, which often limits 3D nanoscopy. Distance-dependent shifts in the fluorescence spectrum^20^ and lifetime^25,26^ can serve as crucial readouts providing an axial ruler with nanometer precision. However, these methods are based on a confocal approach with all its limitations. Here, mirror-enhanced *d*STORM can provide an essential tweak to improve the spatial resolution of 3D-SMLM. Mirror-enhanced *d*STORM holds another unique asset with respect to 3D-SMLM: it not only boosts resolution but also reduces the required laser power to 50% while preserving the desirable blinking behavior. Importantly, for our two-dimensional nanocoating, there are no localization artifacts that arise from emitter-nanostructure coupling. This is in contrast to the well-known situation for zero-dimensional and one-dimensional nanostructures^27–29^, where the emitter-nanostructure “asymmetry” induces signal distortions due to coupling between the emitter’s electromagnetic field and the nanostructure.

Beyond SMLM, the method can be used to enhance the performance of various established fluorescence techniques^30^. With respect to high-content imaging and lab-on-the-chip approaches, mirror-enhanced SMLM outperforms TIRF-based illumination schemes, where realizing homogeneous illumination over a large field of view is still challenging^31,32^.

## Electronic supplementary material

Supplementary Material

## References

[1] Sauer M, Heilemann M (2017). Single-molecule localization microscopy in eukaryotes. Chem. Rev..

[2] Bailey B, Farkas DL, Taylor DL, Lanni F (1993). Enhancement of axial resolution in fluorescence microscopy by standing-wave excitation. Nature.

[3] Drexhage KH, Kuhn H, Schäfer FP (1968). Variation of the fluorescence decay time of a molecule in front of a mirror. Ber. Bunsen-Ges. Phys. Chem..

[4] Le Moal E (2007). Enhanced fluorescence cell imaging with metal-coated slides. Biophys. J..

[5] Drexhage KH (1974). Interaction of light with monomolecular dye layers. Prog. Opt..

[6] Chance RR, Prock A, Silbey R (1978). Molecular fluorescence and energy transfer near interfaces. Adv. Chem. Phys..

[7] Yang XS (2016). Mirror-enhanced super-resolution microscopy. Light Sci. Appl..

[8] Betzig E (2006). Imaging intracellular fluorescent proteins at nanometer resolution. Science.

[9] Rust MJ, Bates M, Zhuang XW (2006). Sub-diffraction-limit imaging by stochastic optical reconstruction microscopy (STORM). Nat. Methods.

[10] Heilemann M (2008). Subdiffraction-resolution fluorescence imaging with conventional fluorescent probes. Angew. Chem. Int. Ed. Engl..

[11] Jungmann R (2010). Single-molecule kinetics and super-resolution microscopy by fluorescence imaging of transient binding on DNA origami. Nano Lett..

[12] Quan TW, Zeng SQ, Huang ZL (2010). Localization capability and limitation of electron-multiplying charge-coupled, scientific complementary metal-oxide semiconductor, and charge-coupled devices for superresolution imaging. J. Biomed. Opt..

[13] Grimm JB (2015). A general method to improve fluorophores for live-cell and single-molecule microscopy. Nat. Methods.

[14] Altman RB (2011). Cyanine fluorophore derivatives with enhanced photostability. Nat. Methods.

[15] Li WX, Stein SC, Gregor I, Enderlein J (2015). Ultra-stable and versatile widefield cryo-fluorescence microscope for single-molecule localization with sub-nanometer accuracy. Opt. Express.

[16] Xu K, Babcock HP, Zhuang XW (2012). Dual-objective STORM reveals three-dimensional filament organization in the actin cytoskeleton. Nat. Methods.

[17] Strambio-De-Castillia C, Niepel M, Rout MP (2010). The nuclear pore complex: bridging nuclear transport and gene regulation. Nat. Rev. Mol. Cell Biol..

[18] Löschberger A (2012). Super-resolution imaging visualizes the eightfold symmetry of gp210 proteins around the nuclear pore complex and resolves the central channel with nanometer resolution. J. Cell Sci..

[19] Göttfert F (2017). Strong signal increase in STED fluorescence microscopy by imaging regions of subdiffraction extent. Proc. Natl. Acad. Sci. USA.

[20] Elsayad K (2013). Spectrally coded optical nanosectioning (SpecON) with biocompatible metal-dielectric-coated substrates. Proc. Natl. Acad. Sci. USA.

[21] Nieuwenhuizen RPJ (2013). Measuring image resolution in optical nanoscopy. Nat. Methods.

[22] Franke C, Sauer M, van de Linde S (2017). Photometry unlocks 3D information from 2D localization microscopy data. Nat. Methods.

[23] Takeda A, Wu JJ, Maizel AL (1992). Evidence for monomeric and dimeric forms of CD45 associated with a 30-kDa phosphorylated protein. J. Biol. Chem..

[24] Cabriel C, Bourg N, Dupuis G, Lévêque-Fort S (2018). Aberration-accounting calibration for 3D single-molecule localization microscopy. Opt. Lett..

[25] Chizhik AI, Rother J, Gregor I, Janshoff A, Enderlein J (2014). Metal-induced energy transfer for live cell nanoscopy. Nat. Photonics.

[26] Karedla N (2018). Three-dimensional single-molecule localization with nanometer accuracy using Metal-Induced Energy Transfer (MIET) imaging. J. Chem. Phys..

[27] Ropp C (2015). Nanoscale probing of image-dipole interactions in a metallic nanostructure. Nat. Commun..

[28] Raab M, Vietz C, Stefani FD, Acuna GP, Tinnefeld P (2017). Shifting molecular localization by plasmonic coupling in a single-molecule mirage. Nat. Commun..

[29] Mack DL (2017). Decoupling absorption and emission processes in super-resolution localization of emitters in a plasmonic hotspot. Nat. Commun..

[30] Schreiber B (2018). Enhanced fluorescence resonance energy transfer in G-pro-tein-coupled receptor probes on nanocoated microscopy coverslips. ACS Photonics.

[31] Douglass KM, Sieben C, Archetti A, Lambert A, Manley S (2016). Super-resolution imaging of multiple cells by optimized flat-field epi-illumination. Nat. Photonics.

[32] Schreiber B, Elsayad K, Heinze KG (2017). Axicon-based Bessel beams for flat-field illumination in total internal reflection fluorescence microscopy. Opt. Lett..

