# Supplementary material for: Sharpening emitter localization in front of a tuned mirror
Source: Light Sci Appl. 2018 Dec 5;7:99. doi: 10.1038/s41377-018-0104-z (PMC6279778; doi:10.1038/s41377-018-0104-z)
Supplement: Supplementary file 1 — Supplementary Material [file 41377_2018_104_MOESM1_ESM.docx]

Supplementary Information

# Sharpening emitter localization in front of a tuned mirror

Hannah S. Heil^1^, Benjamin Schreiber^1^, Ralph Götz^2^, Monika Emmerling^3^, Marie-Christine Dabauvalle^4^, Georg Krohne^4^, Sven Höfling^3,5^, Martin Kamp^3^, Markus Sauer^2^, Katrin G. Heinze^1,^*

^1^ Rudolf Virchow Center, Research Center for Experimental Biomedicine, University of Würzburg, Josef-Schneider-Str.2, 97080 Würzburg, Germany

^2^ Department of Biotechnology and Biophysics, Biozentrum, University of Würzburg, Am Hubland, 97074 Würzburg, Germany

^3^ Technische Physik, Physikalisches Institut and Wilhelm Conrad Röntgen-Center for Complex Material Systems, University of Würzburg, Am Hubland, 97074 Würzburg, Germany

^4^ Division of Electron Microscopy, Biozentrum, University of Würzburg, Am Hubland, 97074 Würzburg, Germany

^5^ SUPA, School of Physics and Astronomy, University of St Andrews, St Andrews, KY16 9SS, United Kingdom

**Materials and Methods**

**Supplementary Figure S1:**

Experimental reproducibility in different sample configurations.

**Supplementary Figure S2:**

Fluorescence intensity saturation of A647

**Supplementary Figure S3:**

Blinking behavior of A647 labeling the pore anchoring protein gp210 in NPCs on coated versus uncoated glass coverslips

**Supplementary Figure S4:**

Mirror-enhanced *d*STORM on microtubules

**Supplementary Figure S5:**

Superresolved dual-color images of immunolabeled CD45 receptors in Jurkat T-cells.

**Supplementary Figure S6:**

Quality control, sample preparation and mounting.

1. **Materials and Methods**
   1. **Substrate fabrication**

The metal-dielectric substrates were fabricated on 170 µm thick glass coverslips (Menzel Coverslip 24x24 mm, #1.5, selected, Fisher Scientific), which were cleaned in piranha-solution (3 parts of 95-98 %H_2_SO_4_ (Roth, X944) and 1 part of 30 % H_2_O_2_ (AppliChem, A0626)) at 70 °C for 20 minutes and dried at 100 °C for 10 minutes or under vacuum conditions for 20 min prior to fabrication. A 2 nm germanium (Ge, 99.999 %, GE006102/1, Goodfellow) wetting layer was deposited by electron beam evaporation followed by a silver (Ag, 99.99 %, AG006105/7, Goodfellow) layer (thickness: 20 nm or 50 nm). Finally, a dielectric spacer layer was applied on top by RF-sputtering of silicon nitrite (Si_3_N_4_, Goodfellow, SJ619300) until a thickness of 28 nm or 10 nm was reached. During deposition the growth rate and layer thickness was monitored with a quartz crystal microbalance. The resulting layer composition was verified by spectroscopic ellipsometry (alpha-SE, J.A. Woollam, US). The average (root mean square) roughness of the silver layer was determined by atomic force microscopy (Molecular Force Probe MFP-3D system, Asylum Research, and AC240TS probes, Olympus) to be 0.53 ± 0.03 nm (see **Supplementary** **Fig. S6a**). The metal-dielectric substrates were stored under vacuum conditions or in ethanol (Sigma, 32205). As a control, plane glass coverslips were used after cleaning by sonicating for 1 hour in chloroform (Sigma, 472476), drying, sonicating for 1 hour in 5 M NaOH (Roth, 6771) solution and washing in ddH2O. The cleaned glass coverslips were stored in ethanol.

- 1. **Nuclear pore complex (NPC) sample preparation and labeling**

Prior to nuclear envelope preparation both, metal-dielectric substrates and glass coverslips were silanized to enhance the attachment of the nuclear membrane to the surface. For this, the metal-dielectric substrates (2 nm Ge, 50 nm Ag and 10 nm Si_3_N_4_) and the glass coverslips were treated for 1 minute with a mild plasma at 0.1 mbar at 15 mA and 115 V and incubated for 12 hours in a 0.01 % 3-Aminopropyltriethoxysilan solution (Sigma, A3648) with 90 % Ethanol (Fisher Cemical, E/0650DF/17), 5 % ultrapure water and 5 % acetic acid (Sigma, A6283) at 4°C before drying them with nitrogen gas. Nuclear envelopes were manually isolated and spread on cover glasses or substrates as described elsewhere^1^. After a 15 minute fixation step in 2 % formaldehyde (Sigma, F8775) in PBS the nuclear envelopes were washed in PBS and blocked for 20 minutes in 5 % BSA (Sigma, A3983) in PBS. For the immunolabeling the nuclear envelopes were incubated for 1 hour with X222 antibodies (provided by Georg Krohne^2^), washed for 10 min in PBS and incubated another hour with A647 F(ab’)_2_ fragments of goat anti-mouse IgG (Life Technologies, A21237). After a final PBS washing step for 20 minutes the nuclear envelopes were incubated in 2 % formaldehyde in PBS for 10 minutes and stored in PBS. Before an experiment, a flow cell was constructed by placing two 2 mm stripes of double sided tape (TESA) along the long axis of a cleaned 170 µm thick 24x40 mm glass coverslip (Menzel Coverslip, Fisher Scientific) and placing the glass coverslip or substrate carrying the nuclear envelopes on top with the envelopes facing inside (**see Supplementary Fig. S6b**).

- 1. **Microtubule sample preparation**

20 µg of lyophilized HiLyte 647 labeled porcine tubulin (tebu-bio, 027TL670M-A) were diluted in 5 µl of the ice-cold polymerization buffer (1 mM GTP (tebubio, BST06), 5 mM MgCl2 (AppliChem, A1036) and 5 % DMSO (AppliChem, A1584) in BRB80) and placed on ice for 5 minutes before incubating the tubulin solution at 37 °C for 2 hours. The microtubules were stabilized by adding 195 µl of 10 µM paclitaxel (tebu-bio, TXD01) in BRB80. After spinning down the microtubules at 16100 x g and 23 °C for 20 minutes the supernatant was discarded and the remaining pellet was resuspended in 200 µl of 10 µM paclitaxel in BRB80. The polymerized and stabilized microtubules were stored at room temperature and used within the next week. Before an experiment, a flow cell was constructed as described in the previous section. The metal-dielectric substrate (2nm Ge, 20 nm Ag and 28 nm Si_3_N_4_) was washed in ethanol and dried with N2 just before the flow cell construction. For the immobilization of the microtubules on the surface, the microtubule solution was diluted 1: 10 in 10 µM paclitaxel in BRB80 and centrifuged for 20 minutes at 16100 x g and 23 °C. The remaining pellet was resuspended in 10 µM paclitaxel in BRB80. 20 µl of the resulting purified microtubule solution was incubated for 10 min in the flow channel. After a 40 µl wash with 10 µM paclitaxel in BRB80, 20 µl of a 1 µM avidin solution (Sigma, A9275) with 10 µM paclitaxel were incubated for 5 minutes in the channel before washing again with 40 µl of 10 µM paclitaxel in BRB80. Finally, the microtubules were fixed with 2 % glutaraldehyde (Merck, 104239) and 10 µM paclitaxel solution for 10 minutes and stored in BRB80.

- 1. **Jurkat T-cell sample preparation and labeling**

Jurkat T-cells were cultured in RPMI 1640 containing 10 % FCS, 2 mM L-glutamine, 100 U ml^-1^ penicillin and 0.1 mg ml^-1^ streptomycin at 37 °C and 5 % CO_2_. The purified anti-human CD45 antibody (BioLegend, 368502) was labeled and purified with 0.5 ml 7 kDa Spin Desalting Columns (Thermofisher, 89882) in accordance with the instruction manual. The antibody labeling was performed in 100 mM NaHCO_3_ solved in PBS with 5 fold molar excess of the NHS-dye (A532 NHS ester or A647 NHS ester, Thermofisher, A20101MP and A37566) by incubation in the dark for 2.5 hours. In a second step the conjugated antibody was purified and the buffer was exchanged through a fresh column with 0.02% NaN_3_ solved in PBS. The conjugated antibody was stored at 4 °C. The degree of labeling of the conjugated antibody was determined with a UV-vis spectrophotometer (Jasco V-650) to ~5. In order to allow the Jurkat T-cells to settle on the surface the coated (2nm Ge, 50 nm Ag and 10 nm Si_3_N_4_) and uncoated 24x24 mm coverslips were treated for 1 minute with a mild plasma at 0.1 mbar at 15 mA and 115 V. 1.8 million cells in culture medium were seeded on the treated coverslip in a 6 well plate and incubated for 20 minutes at 37 °C and 5 % CO_2_. After a 5 minute incubation on ice the culture medium was removed and the cells were labeled for 30 min in PBS containing 5 µg ml^-1^ of CD45 antibody conjugated with A532 and 5 µg ml^-1^ of CD45 antibody conjugated with A647. Following this the cells were washed three times with PBS, fixed for 15 minutes in 4% formaldehyde and 0.25 % glutaraldehyde (Sigma, G5882) in PBS, and finally stored in PBS after a last step of PBS washing. Before an experiment, a flow cell was constructed as described previously.

- 1. **Microsphere labeling and sample preparation**

Biotin coated 15 µm microspheres (Kisker Biotech, PC-BX-15.0) were labeled with Alexa647 functionalized with streptavidin as described elsewhere^3^. To place the fluorescently labeled microspheres on the nanocoating or glass surface the microsphere solution was incubated in a flow cell (construction described in section 1.2) for 20 minutes with the 24x24 mm glass coverslip or nanocoated coverslip (2nm Ge, 50 nm Ag & 10 nm SiN) facing downwards. Before mounting the sample on the microscope the flow cell was carefully flushed with imaging buffer.

- 1. **Cos7 labeling and sample preparation**

Cos7 cells were cultured in Full Growth Media (DMEM containing 10 % FCS, 2 mM L-glutamine, 100 U ml^-1^ penicillin and 0.1 mg ml^-1^ streptomycin) at 37 °C and 5 % CO_2_ and harvested by scraping. 200 000 cells were seeded on 24x24 mm glass coverslips or coated coverslips (2nm Ge, 50 nm Ag & 10 nm SiN) and incubated at 37 °C and 5 % CO_2_ in Full Growth Media for 2 hours. Following this cells were fixed and permeabilized for 2 minutes in cytosceleton buffer (10 mM MES, 150 mM NaCl, 5 mM EGTA, 5 mM glucose, 5 mM MgCl_2_ in ultrapure water) containing 0.3 % (v/v) glutaraldehyde (Sigma, G5882) and 0.25 % (v/v) Triton-X (Roth, 3051.3) at 37 °C and for 10 min in cytoskeleton buffer containing 2 % (v/v) glutaraldehyde at 37 °C. Glutaraldehyde autofluorescence was quenched by a 7 minute incubation in fresh 0.5 % NaBH_4_ in PBS refreshing the solution twice during incubation. Next cells were washed with PBS with a sequence of 1 minute, 5 minutes and 10 minutes washing steps and blocked for 30 minutes with 5 % BSA in PBS. Immunolabeling was performed by one hour incubation at room temperature with 10 µg ml^-1^ monoclonal mouse anti-β-tubulin antibody (Sigma, T8328) and 5% BSA in PBS, followed by two 5 minute washing steps with 1 % Tween-20 (Sigma, 93774) in PBS, two 5 minute washing steps with PBS and 1 hour incubation at room temperature with 10 µg ml^-1^ A647 F(ab’)_2_ fragments of goat anti-mouse IgG. Finally the cells were washed with 0.1 % Tween-20 in PBS for 1 minute and twice for 5 minutes, twice for 5 minutes with PBS and a postfixation was performed for 10 minutes in 2 % formaldehyde in PBS, followed by washing the cells three times for 10 minutes with 1 % Tween-20 in PBS. Before an experiment, a flow cell was constructed as described previously.

- 1. ***d*STORM imaging**

The *d*STORM imaging was performed with an inverted light microscope (Zeiss Observer Z.1, Carl Zeiss AG) equipped with an EMCCD camera (iXon ultra DU-897U-CSO-#BV, Andor), a C-APOCHROMAT 1.15 NA, x63 water objective (LD C-Apochromat 63x NA 1.15, 421887-9970, Carl Zeiss) and an optovar with 2.5-fold magnification. For TIRF and EPI illumination configuration an A-Plan Apochromat 1.46 NA, 100x oil objective (A-Plan Apochromat 100x NA 1.46, 420792-9800, Carl Zeiss) with a 1.6-fold magnification optovar was used. The excitation was performed with a 640 nm laser (iBEAM-smart-640-S, Toptica) and a 532 nm laser (GEM 532 nm, 250 mW, Laser Quantum) through an illumination lens with 40 mm focal length, resulting in an effective illumination intensity of 1-5 kW cm^-2^. For TIRF illumination the In the excitation light path a clean-up filter (640/8 nm MaxDiode™ laser clean-up, Semrock) is passed before a dichroic mirror (BrightLine quadedge 405/488/532/635, Semrock) separates excitation and emission light. In the detection path an additional notch filter (StopLine quadnotch ZET 405/488/561/647, Semrock) and a bandpass filter (700/75 ET, Chroma) are placed. For dual-color imaging the fluorescence was split with an Longpass (Edge Basic 635 LP, Semrock) to a second EMCCD camera of the same type with an additional bandpass filter (BrightLine HC 582/75, Semrock). After applying the *d*STORM buffer (125 mM MEA (Sigma, M6500), 20 mM D-glucose (Sigma, G7528), 0.55 mg ml^-1^ gluco-oxidase (Roth, 60281), 0.011 mg ml^-1^ catalase (Sigma, C1345) in PBS adjusted to pH 7.9 with 1M KOH (Sigma, 30603) solution) image stacks of 20000 frames were acquired with an exposure time of 5 ms. In case of the *d*STORM experiments with the microspheres and the Cos7 cells the exposure time was 10 ms. For the dual-color experiments the *d*STORM buffer contained 100 mM MEA at a pH of 7.7 and the 10000 frames were acquired at a exposure time of 10 ms. The excitation power was reduced to 30 % for the experiments on coated substrates. In order to allow two channel alignment for the dual-color imaging calibration images with 100 nm TetraSpeck microspheres in water immobilized on glass coverslips were recorded simultaneously in both color cannels. For the microsphere experiment additional images at the equatorial plane of each sphere where acquired at low laser power 1.5 W cm^-2^ and with an exposure time of 100 ms.

- 1. **Image reconstruction and analysis**

*d*STORM images were reconstructed with the ImageJ plugin ThunderSTORM ^4^. For each set of experiments, the same localization parameters were used. In a first step drift correction via the cross correlation method with 15 bins and a magnification factor of 5 was performed in case of the NPC data. For the NPC reconstruction, only localizations with an uncertainty of more than 3 nm and less than 30 nm were considered. Localization duplicates were filtered by merging localization events that repeatedly occurred within a radius of 15 nm with a maximum of 50 off-frames in between and in order to discard events from unspecific background a density filter of a minimum of 5 events in a radius of 50 nm was applied. Based on the filtered localization data a superresolved image with a pixel size of 5 nm was reconstructed by Gaussian rendering. The FRC analysis was performed on the filtered localization data used for image reconstruction with the ImageJ plugin FIRE^5^. The localization data was further analyzed with the ImageJ plugin TRABI^6^ with an aperture radius of 7.5 pixels, 7 frames for the background characterization with a basejump of 2 frames, an exclusion zone with a radius of 14 pixels and a highlander filter of 100 frames. The reoccurrence of each event was analyzed with the ImageJ plugin ThunderSTORM^4^ based on the drift corrected localization data by merging events within a radius of 15 nm, while allowing 5000 off frames. These parameters were chosen due to their robustness. For the microtubule data the localization and TRABI analysis was performed accordingly with few exceptions: drift correction was not necessary and only localizations with less than four neighbors in a 60 nm radius were discarded. The reoccurrence was analyzed within a radius of 20 nm, while allowing 500 off frames. The dual-color localization analysis was performed with RapidSTORM^7^ in order to allow two channel alignment of the localization data to correct shifts due to chromatic aberrations. For the channel alignment the TetraSpeck images were localized and a superresolved image with a pixelsize of 10 nm was reconstructed for each channel. Based on this calibration images a transformation matrix of the A532 channel onto the A647 was calculated with the ImageJ plugin bunwarpJ^8^. This transformation matrix was directly applied with the RapidSTORM localization analysis of the A532 data. Localizations were filtered to exclude events with a photon count exceeding 20000 photons, a localization uncertainty higher than 50 nm and with less than 5 neighbors in a 50 nm radius. Based on this data superresolved images with a pixel size of 10 nm were reconstructed by Gaussian rendering. In case of the micosphere data the radius and center of each sphere were calculated based on the images of the equatorial plane. The *d*STORM image series were localized with ThunderSTORM, localizations with an intensity above 5000 photons and a localization uncertainty below 3 nm or above 30 nm were discarded. As described by Cabriel et al.^3^ the z-position of each localization was calculated based on the sphere radius and the radial position ρ of the localization with respect to the center.

For the Cos7 data the multi-emitter fitting option of ThunderSTORM was used, localizations with an intensity below 100 and above 5000 photons and an uncertainty below 5 nm and above 50 nm were discarded. Also the data was corrected for drift and a density filter was applied as described above. The localization data was binned in 20 nm pixel bins, for each bin the median uncertainty was calculated and the resulting matrix was illustrated as a colormap.

- 1. **Simulation**

Simulations of the enhancement effect based on finite element method calculations were performed with the commercial software Comsol Multiphysics™ 4.4. For the excitation enhancement$F_{I_{\mathrm{ex}}}$, a plane wave excitation encountering the substrate interface was simulated (see **Fig. 3 a)**. Based on the resulting amplitude of the electric fields in the presents ($E_{\mathrm{mod}}$) and absents ($E_{0}$) of the metal-dielectric coating the height dependent excitation intensity enhancement was calculated (see **Fig. 3 b)**:

|  | $F_{I_{\mathrm{ex}}}(h)=\frac{\left\vert E_{\mathrm{mod}}(h) \right\vert^{2}}{\left\vert E_{0}(h) \right\vert^{2}}$ | (1) |
| --- | --- | --- |

To theoretically predict the emission enhancement, the distance dependent radiative decay rates of a dipole located near a metal-dielectric substrate of a glass coverslip were compared. As the fluorophores are expected to be oriented randomly in the experiment the rates of parallel ($\kappa_{\parallel}$) and perpendicular ($\kappa_{\perp}$) dipoles were averaged:

|  | $\kappa_{\mathrm{iso}}=\frac{1}{3}\kappa_{\perp}+\frac{2}{3}\kappa_{\parallel}$ | (2) |
| --- | --- | --- |

The emission enhancement $F_{\mathrm{Em}}$ is based on two effects (see **Fig. 3 e,f**), the modification of the quantum yield $F_{\eta}$ and of the detectability $F_{\det}$ within the collection angle of the detection optics in the vicinity of the metal-dielectric surface:

|  | $F_{\mathrm{Em}}(h)=F_{\eta}(h)\cdot F_{\det}(h)$ | (3) |
| --- | --- | --- |

Besides the radiative decay rates in presents ($\kappa_{r, mod}$) and absents ($\kappa_{r,0}$) of the nanocoating and the non-radiative decay rate $\kappa_{abs,mod}$, $F_{\eta}$ also depends on the intrinsic quantum yield $\eta_{0}$ of the emitter^9^:

|  | $F_{\eta}(h)=\frac{\kappa_{r, mod}(h)/\kappa_{r,0}(h)}{\kappa_{r, mod}(h)/\kappa_{r,0}(h) +\kappa_{abs,mod}(h)/\kappa_{r,0}(h) +(1-\eta_{0})/\eta_{0}}\cdot\eta_{0}^{-1}$ | (4) |
| --- | --- | --- |

The mirror effect of the metal-dielectric coating strongly modifies the dipole emission pattern (see **Fig. 3 c,d**) and leads to a change in the detection efficiency. This can be described by the ratio of the share of the far field emission within the detection angle $\kappa_{NA}$ with and without the coating:

|  | $F_{\det}(h)=\frac{\kappa_{NA,mod}(h)/\kappa_{r,mod}(h)}{\kappa_{NA,0}(h)/\kappa_{r,0}(h)}$ | (5) |
| --- | --- | --- |

Simulations of the fluorescence images of the NPC were calculated with MatLab (Mathworks Inc.) based on single emitters placed on an 8-element ring blurred with 2D-Gaussians with standard deviations resembling the experimentally determined localization uncertainty.

1. **Supplementary Figures**

| 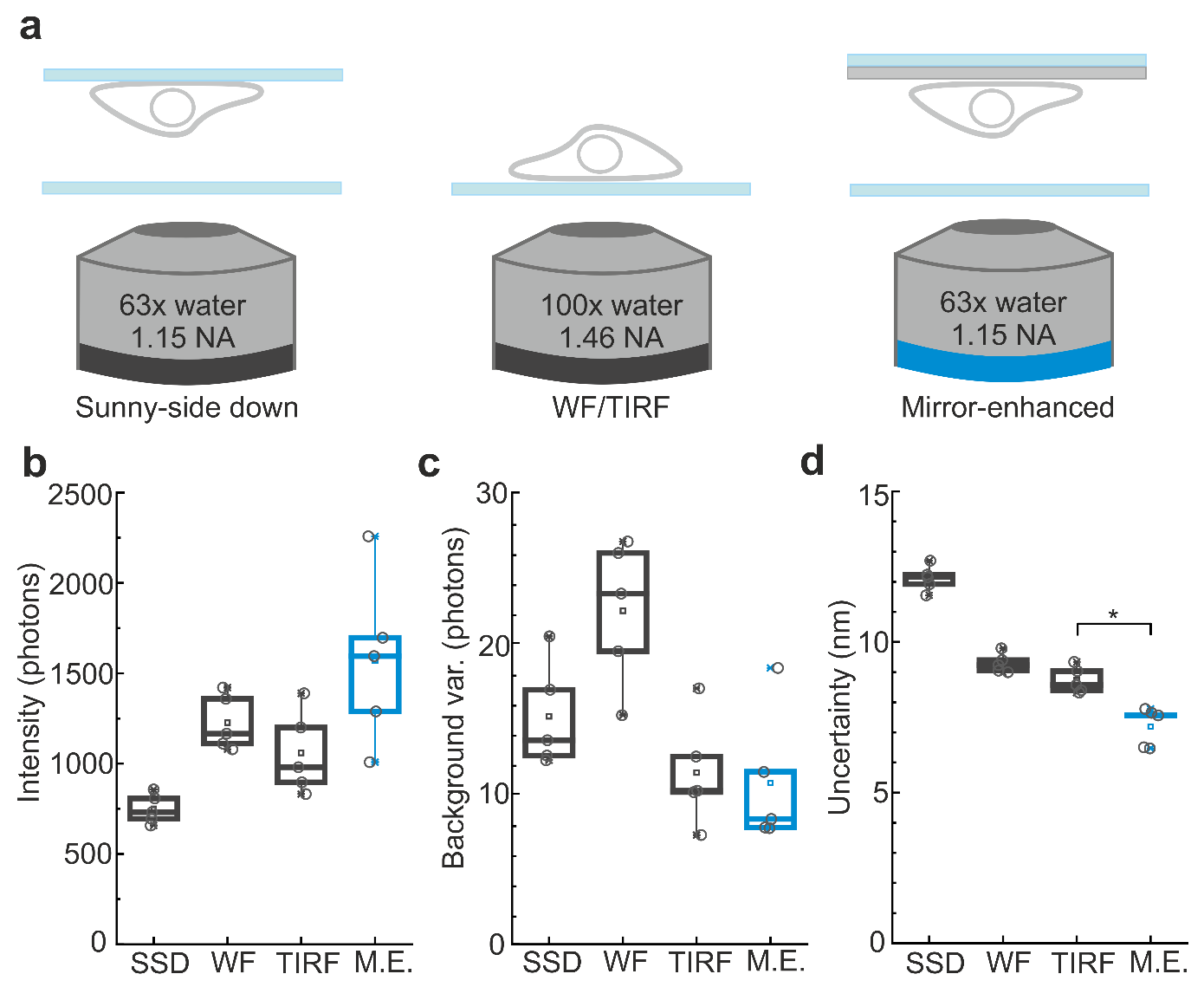 |
| --- |
|  |
| **Supplementary Figure S1**\| Experimental reproducibility in different sample configurations. (**a**) Illustration of the sample configurations. (**b-d**) Boxplots of the intensity. (**b**), the background variance (**c**) and the localization uncertainty (**d**) for sunny-side-down (SSD), widefield (WF), TIRF, and mirror-enhanced *d*STORM (M.E.). |

| 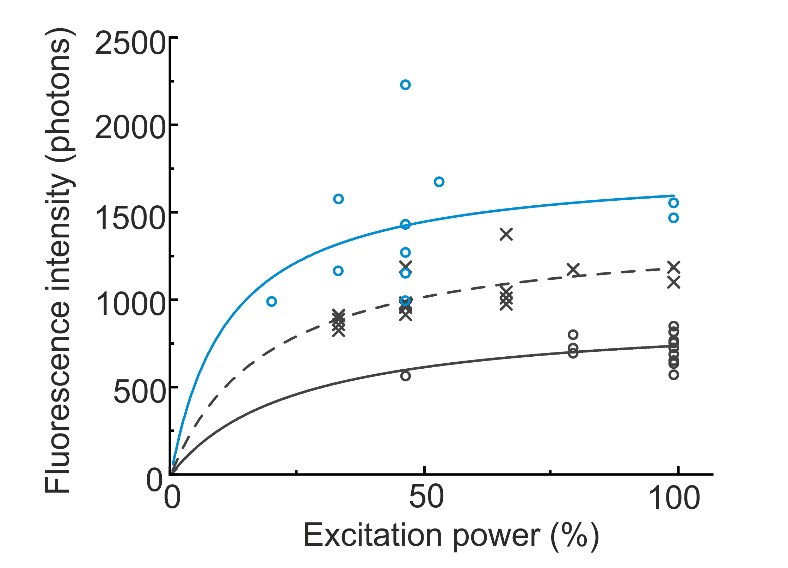 |
| --- |
|  |
| **Supplementary Figure S2**\| Fluorescence intensity saturation of A647 in vicinity of the metal-dielectric coating (blue circles, fit: blue line), in TIRF configuration (gray cross, fit: dashed gray line) and in SSD configuration (gray circles, fit: solid gray line). |

| 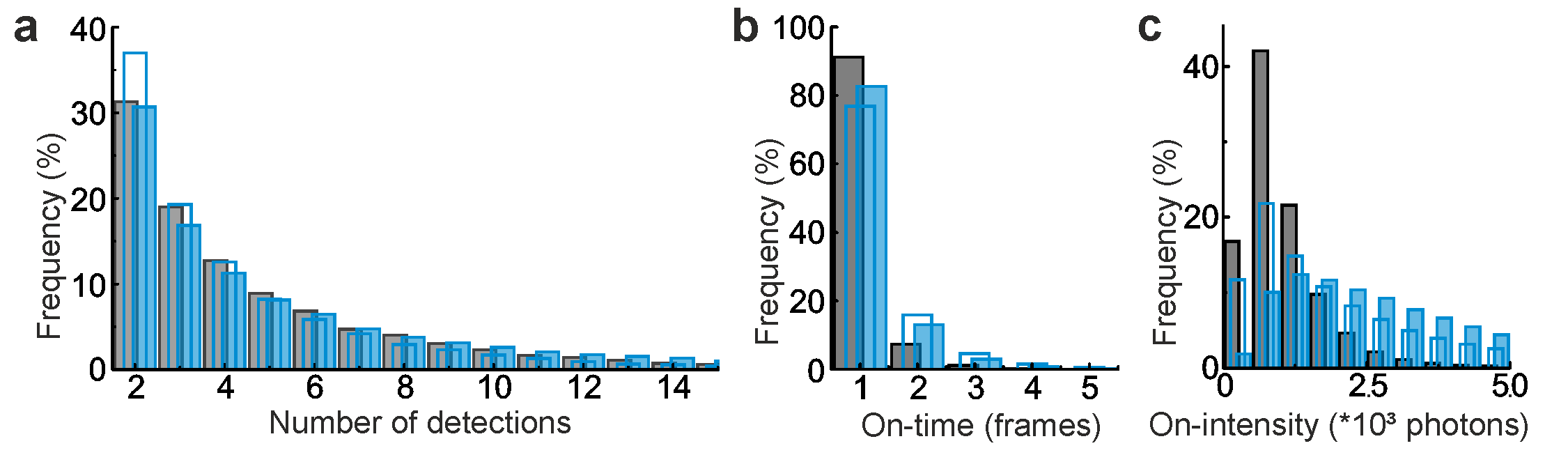 |
| --- |
|  |
| **Supplementary Figure S3**\| Blinking behavior of A647 labeling the pore anchoring protein gp210 in NPCs. Histograms of (**a**) the number of detections (same molecule) (**b**) the duration and (**c**) the total intensity of each on-event for the experiment with the metal-dielectric coated coverslip at 50 % (blue, filled bars), and 100% of the excitation power (blue, open bars) and for the respective experiment on a pure glass coverslip (gray). |

| 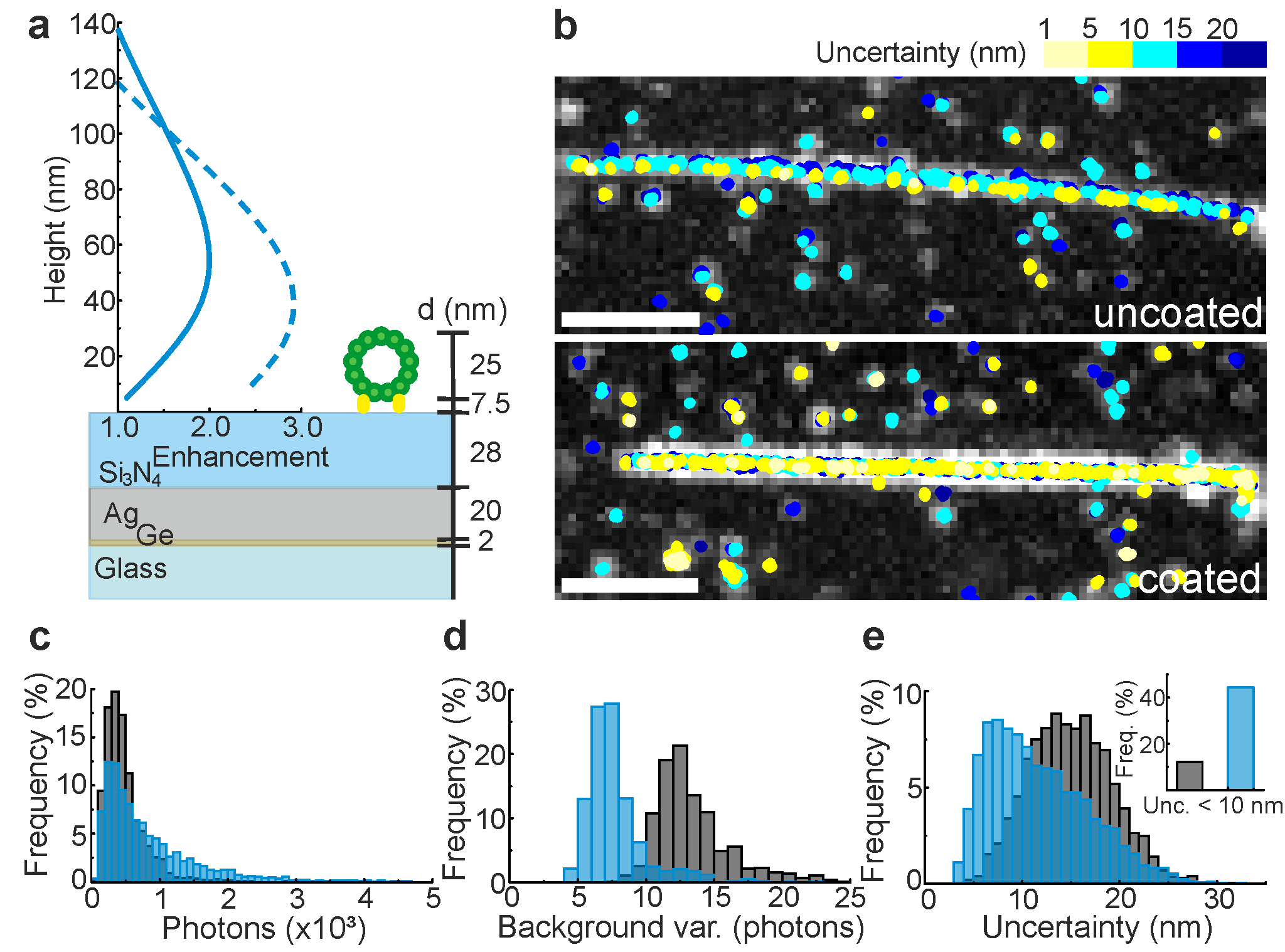 |
| --- |
|  |
| **Supplementary Figure S4**\| Microtubule imaging *in vitro*. (**a**) Experimental setup and simulated height dependent enhancement for HiLyte 647 labeled microtubules immobilized on a metal-dielectric substrate. (**b**) Microtubule images of color coded single HiLyte 647 dye molecules representing the localization uncertainty on an uncoated versus coated coverslip showing a clear signal enhancement on metal-dielectric coatings, (**c-e**) Histograms of the (**c**) intensity distribution, (**d**) the background variance, and (e) the localization uncertainty of the detected events on uncoated (gray) and coated coverslips (blue). The inset in (**e**) highlights the increased number of events with a localization uncertainty below 10 nm for coated (blue) versus uncoated (gray) coverslips. The signal enhancement and reduced noise result in a localization precision enhancement of more than twofold. Scale bars 2 µm. |

| 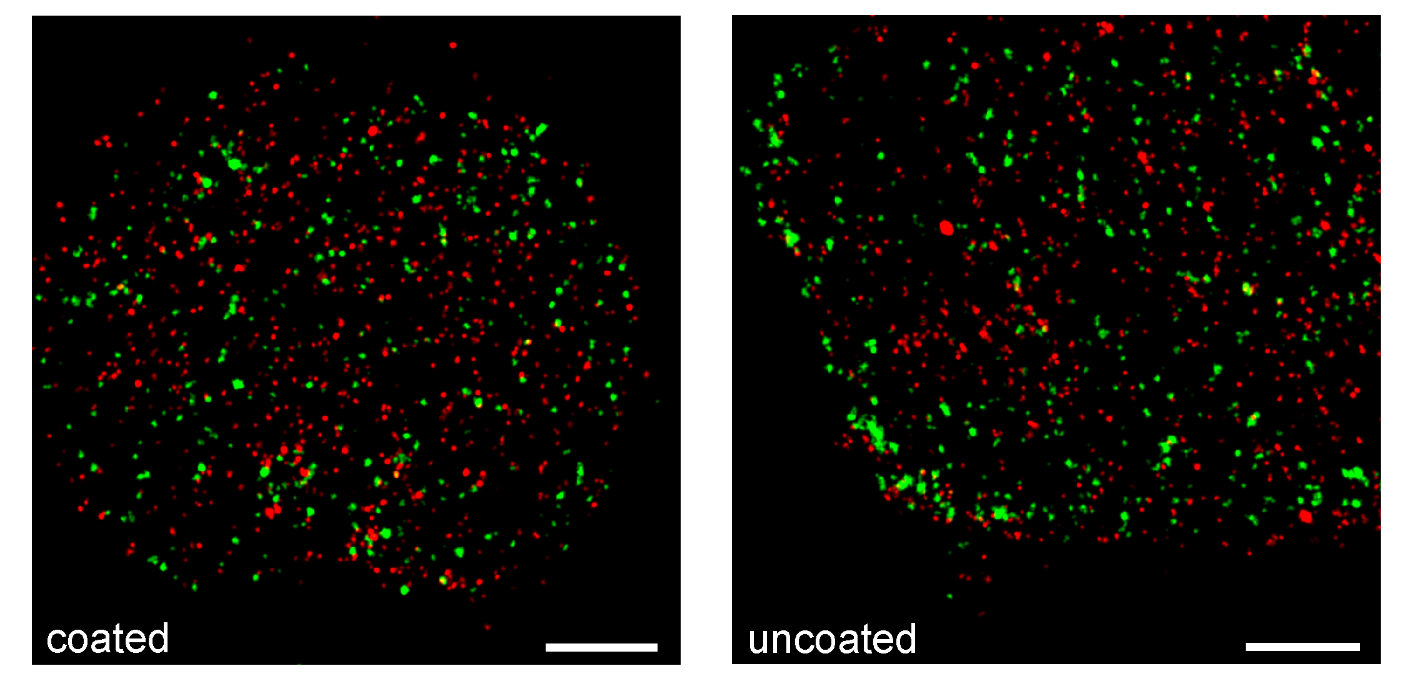 |
| --- |
|  |
| **Supplementary Figure S5**\| Superresolved dual-color images of immunolabeled CD45 receptors in Jurkat T-cells on coated and uncoated glass coverslips. Cells were labeled with a 50:50 mixture of A532 (green) and A647 (red) anti-CD45-antibodies, and thus do not colocalize due the monomeric receptors. Scale bars 2 µm. |

| 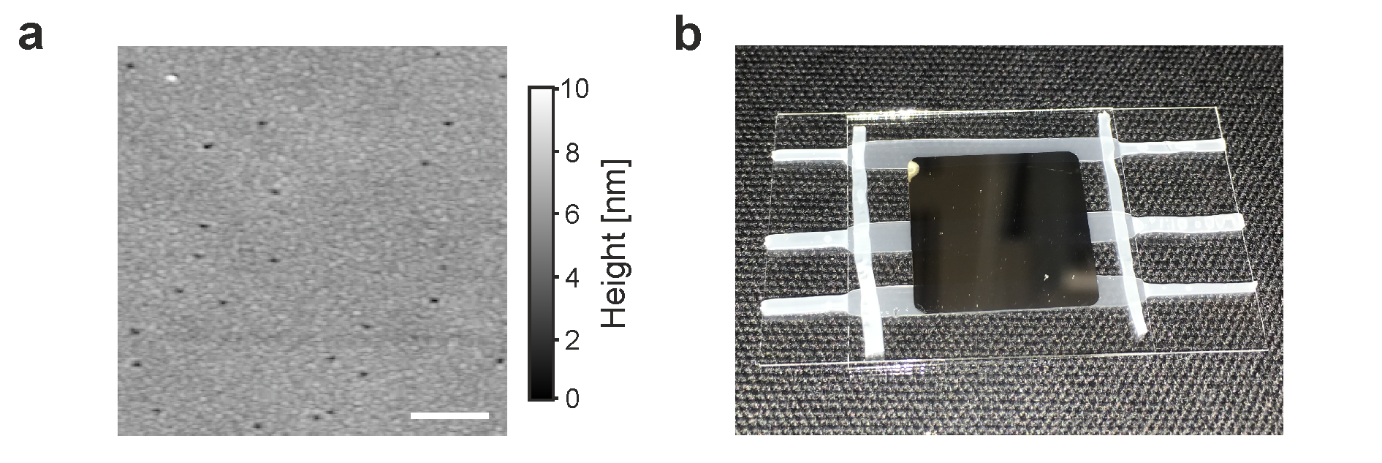 |
| --- |
|  |
| **Supplementary Figure 6**\| Quality control, sample preparation and mounting. (**a**) Surface topography of a silver nanocoating measured by atomic force microscopy (AFM), the average root mean square roughness is 0.54 ± 0.03 nm. (**b**) Flow cell construction. Scale bar 1 µm. |

**References**

1 Löschberger A, Van De Linde S, Dabauvalle MC, Rieger B, Heilemann M *et al.* Super-resolution imaging visualizes the eightfold symmetry of gp210 proteins around the nuclear pore complex and resolves the central channel with nanometer resolution. *J Cell Sci* 2012; **125**: 570–575.

2 Gajewski A, Lourim D, Krohne G. An antibody against a glycosylated integral membrane protein of the Xenopus laevis nuclear pore complex: a tool for the study of pore complex membranes. *Eur J Cell Biol* 1996; **71**: 14–21.

3 Cabriel C, Bourg N, Dupuis G, Lévêque-Fort S. Aberration-accounting calibration for 3D single-molecule localization microscopy. *Opt Lett* 2018; **43**: 174–177.

4 Ovesný M, Křížek P, Borkovec J, Svindrych Z, Hagen GM. ThunderSTORM: a comprehensive ImageJ plug-in for PALM and STORM data analysis and super-resolution imaging. *Bioinformatics* 2014; **30**: 2389–2390.

5 Nieuwenhuizen RPJ, Lidke K a, Bates M, Puig DL, Grünwald D *et al.* Measuring image resolution in optical nanoscopy. *Nat Methods* 2013; **10**: 557–62.

6 Franke C, Sauer M, van de Linde S. Photometry unlocks 3D information from 2D localization microscopy data. *Nat Methods* 2017; **14**: 41–44.

7 Wolter S, Löschberger A, Holm T, Aufmkolk S, Dabauvalle M-C *et al.* rapidSTORM: accurate, fast open-source software for localization microscopy. *Nat Methods* 2012; **9**: 1040–1042.

8 Arganda-Carreras I, Sorzano COS, Marabini R, Carazo JM, Ortiz-de-Solorzano C *et al.* Consistent and Elastic Registration of Histological Sections using Vector-Spline Regularization. In: *Computer Vision Approaches to Medical Image Analysis*. 2006, pp 85–95.

9 Bauch M, Toma K, Toma M, Zhang QW, Dostalek J. Plasmon-Enhanced Fluorescence Biosensors: a Review. *Plasmonics* 2014; **9**: 781–799.
